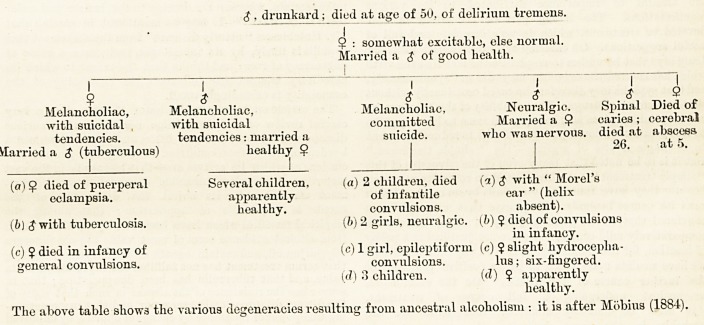# Progress in Psychiatry

**Published:** 1900-02-17

**Authors:** 


### PROGRESS IN PSYCHIATRY.

(*Continued from page* 213.)

**Aetiology and Morbid Heredity.**—(*f*) As regards sex, the various conditions which influence the development of insanity seem to be operative in nearly equal proportion in the two sexes. The result is that nearly equal numbers of the two sexes become subject to mental disease, the preponderance of numbers being slightly on the female side. This preponderance is greater than the relatively small predominance in numbers of the female over the male general population. The particular ætiological factors determining insanity in women, such as the special conditions attendant on child-birth and the puerperium, lactation, unsatisfied sexual life, the menopause, &c., may be balanced against those causes which act more potently on the other sex, such as the rivalries and anxieties of business and of professional life, the freer indulgence in alcoholic liquors and other excesses, and the greater liability to cranial and cerebral injuries and accidents. The incidence of syphilis also appears to be fraught with more serious consequences as regards cerebral and mental involvement when it occurs in the male than when it affects the female. The combination, however, of the effects of syphilis and of alcohol on a considerable proportion of the female population in great cities, viz., the immoral and prostitute class, undoubtedly serves to enhance the proportion of female victims to cerebral and mental disease. In various localities these factors must vary, and hence the figures and lunacy statistics furnished by different counties in the United Kingdom show corresponding differences.

(*g*) The proportion of insane (including in this category imbeciles and idiots) to normal or sane individuals is about 1 to 300 of the general population for the United Kingdom. The total number of lunatics in England and Wales on January 1st, 1899, was 105,086; in Scotland it amounted to 15,400; and in Ireland to 20,304. The official returns published by the Commissioners in Lunacy for England and Wales show the following ratios of insanity: In 1869 it was 1 to 418 of the population; in 1879 it was 1 to 363; in 1889 it was 1 to 337; and in 1899 it was 1 to 302. In Scotland the ratios were as follows: In 1881 it was 1 to 374 of the general population; in 1891 it was 1 to 320; and in 1899 it was 1 to 278. The corresponding figures for Ireland were as follows: In 1871 it was 1 to 328; in 1881 it was 1 to 281; and in 1899 it was 1 to 222. It is probable that an increase in some of the factors capable of producing insanity before-mentioned, such as alcoholism, syphilis, and the overstrain and excessive wear and tear of modern life is in large measure responsible for the significant and sinister increase of insanity amongst the general population during the last few decades which the above figures indicate. It will take probably another decade of careful observation and inquiry before the actual rate of the progress of insanity in the British Isles can be exactly ascertained, but a closer and clearer estimate seems to be obtainable year by year.

**Laws of Heredity.**—The so-called laws of heredity are generalisations drawn and formulated from a vast body of facts observed mostly in the domain of common experience. In regard to disease the laws of pathological heredity are the outcome of special but limited observation. Some of these laws can boast of a respectable antiquity, while others are of recent date. In the special department of cerebral and mental disease the laws of heredity were first established on an adequate and scientific basis by Morel[8] in 1860 in a work of classical importance. This branch of investigation has received much attention at the hands of clinical observers mainly of the French school. Very interesting contributions have been made by Dejerine,[9] Legrain,[10]

Möbius,[11] and Féré[12]; while the School of Criminal Anthropologists and of Sociologists, represented by Lombroso,[13] Dugdale,[14] Bruce Thompson[15] and others, has given us much valuable information on cognate and collateral subjects. A careful study of the data enables the following general laws of heredity to be enunciated:—

1. The child tends to inherit the fundamental attributes or qualities of both parents (law of similarity).
2. The qualities inherited from one parent may preponderate (law of prepotency).
3. Qualities strongly marked in both parents tend to be inherited in a very marked degree (law of cumulative or convergent heredity).
4. Qualities in the parent may not appear in the offspring (latent heredity), but may reappear in the next generation or later still (reversion or atavism).
5. Inherited qualities, *cæteris paribus*, will appear in the offspring at the same time at which they became manifest in the parents (law of homochronous heredity), or if intensified by cumulative heredity they may appear precociously.
6. Morbid conditions or diseases which are hereditarily transmitted may produce in the offspring similar conditions (similar morbid heredity), or dissimilar conditions having nevertheless a deep-seated affinity to the parental condition (heredity by transformation).

The following observations will serve to illustrate and explain some of the facts embodied in the above six laws of heredity: Plants produce seed and seedlings, and animals produce young "after their kind." And men "do not gather grapes of thorns, nor figs from thistles." The law that every species produces its kind and every pair its own specific and peculiar offspring is the outcome of facts which by their countless number, constancy, and uniformity have acquired in our mind almost the aspect of necessity. This idea of heredity was expressed by the ancients under the picture of Fate presiding over human destinies. It must be remembered, on the other hand, that variation, as Darwin was the first to point out and to demonstrate, also plays a part in determining the final characteristics of the offspring, and it must not be assumed that the law of heredity holds for all the small and trivial details of structure and attribute manifested in the offspring. Instances, however, do occur in which apparently even trifling incidents serve to illustrate the potency of heredity. Peculiarities in gait and gesture are often inherited, handwriting has been well known to exhibit similar features and peculiarities in parents and offspring, while occasionally some feature in facial appearance appears so marked throughout several generations as to become notorious; and in this connection reference may be made to what are now matters of historical knowledge, viz., the characteristic Napoleonic features, the Bourbon nose, and the Hapsburg lip, in several generations of the French and Austrian Imperial families, which appeared in male members of the family. The transmission of such characters illustrates the law of prepotency on the male side, just as much as the hereditary transmission of hæmophilia illustrates a peculiarity appearing in males only, but transmissible by both sexes. The tendency to suicide has a fatalistic heredity; children at the age of five have been known to exhibit it. Esquirol relates a case in which the grandfather, father, and son all committed suicide at about their fiftieth year. Hammond[16] relates the case of a man who, at the age of 35, cut his throat with a razor while in a bath. He had three children, two sons and a daughter. The two sons committed suicide in the same manner and at the same age as the father, and the daughter, at the age of 34, also cut her throat in a bath. She left behind her a child, who, when she grew up, attempted twice to commit suicide, and succeeded the third time, at age of 31, in killing herself in the same way as her mother did. The operation of reversion or atavism, is met with in some cases of idiocy, of imbecility, and of allied forms of degenerative insanity. Occasionally, under certain conditions, an individual of one sex will assume the appearances of the opposite sex. Women at the climacteric are known occasionally to develop a beard and a masculine voice, the cessation of the sexual life bringing with it a reversion to some remote ancestral type. "There is in every asylum," says Dr. Mercier, "a certain number of bearded and bass-voiced women, whose insanity is usually of a very intractable type; and I have had under care at the same time two men whose hairless face, large mammæ, and shrill voices betokened an assumption of the secondary characters of the other sex, and whose insanity was notably intractable."[17]

It was formerly supposed that close blood relationship of the parents was competent to produce insanity in the offspring. This, however, appears to be disproved by more recent observers. The history of the Ptolemies also affords light in this connection. Among the Ptolemies of Egypt the king always married his sister, yet there was no marked insanity in the dynasty during a period of 300 years. The amount of in-breeding was certainly extreme and exceptional in this instance, but it was not so close as to seriously damage the stock. "Manifest evil," says Darwin, "does not follow from pairing the nearest relations (among animals) for two, three, and even four generations." From the records of the Ptolemy family, it appears that there was a crossing with fresh blood, which broke the continuity of in-breeding, once every three or four generations. The real danger which is likely to result from consanguinity is that which may result from the operation of law number (3) before enunciated, viz., that that qualities strongly marked in both parents tend to be inherited in a very marked degree (law of cumulative or convergent heredity). Under the operation of this law similar morbid tendencies, if present in both parents—as would be likely to be the case in close blood-relations inheriting from a common parent or grandparent—would tend to be intensified to such a degree as to bring about grave degeneracy in the offspring. And this is a fact of importance, because defects and disorders such as deaf-mutism, congenital blindness, and similar conditions are well known to result from the inter-marriage of close blood-relations, especially if the taint (of deaf-mutism, &c.) appears in one of the parents.

The following genealogical tables will serve to throw varied and incidental lights on the various points regarding ætiology and heredity already touched upon, as well as to illustrate the practical conclusions to be drawn :—

1st Generation : ♂ Weak. = ♀ Hysterical.

| 2nd Generation : | ♂ Drunkard. | ♂ Drunkard, debauched. | ♂ Inveterate drunkard. = ♀* Alcoholic and hysterical. |
|---|---|---|---|

| ♂ | ♂ | ♀ | ♀ | | | ♀ |
|---|---|---|---|---|---|---|
| Very nervous, of passionate disposition. | Epileptic. | Extremely nervous. | Feeble, partially deaf, with lisping speech. | Imbecile, convulsions, vicious instincts. | Several (10) children, all delicate, and died young. | Hysterical, Married : Nine children, of whom several died of convulsions. |

* The above shows the result of alcoholic heredity (which is bilateral and convergent), and complicated with hysteria. The table is after Legrain (1895).

♂, drunkard ; died at age of 50, of delirium tremens.

♀ : somewhat excitable, else normal. Married a ♂ of good health.

| ♀ | ♂ | ♂ | ♂ | ♂ | ♀ |
|---|---|---|---|---|---|
| Melancholiac, with suicidal tendencies. Married a ♂ (tuberculous) | Melancholiac, with suicidal tendencies : married a healthy ♀ | Melancholiac, committed suicide. | Neuralgic. Married a ♀ who was nervous. | Spinal caries ; died at 26. | Died of cerebral abscess at 5. |
| (a) ♀ died of puerperal eclampsia. (b) ♂ with tuberculosis. (c) ♀ died in infancy of general convulsions. | Several children, apparently healthy. | (a) 2 children, died of infantile convulsions. (b) 2 girls, neuralgic. (c) 1 girl, epileptiform convulsions. (d) 3 children. | (a) ♂ with "Morel's ear" (helix absent). (b) ♀ died of convulsions in infancy. (c) ♀ slight hydrocephalus ; six-fingered. (d) ♀ apparently healthy. | | |

The above table shows the various degeneracies resulting from ancestral alcoholism : it is after Möbius (1884).

Morel in his treatise already referred to gives the following history of a family for three generations as illustrating the results of alcoholism :—

First Generation.—Father, a drunkard.

Second Generation.—Son, a drunkard. Was disgustingly drunk on his marriage day.

Third Generation.—Seven grandchildren. The first and second died of convulsions ; the third was an idiot at 22 years of age ; the fourth was melancholic with suicidal tendencies, and became a dement ; the fifth was not insane, but was peculiar and irritable ; the sixth had had repeated attacks of insanity ; the seventh was nervous and depressed and used to utter the most despairing misgivings as to his life and reason.

A very interesting and instructive chapter is published by Dr. W. Ireland,[18] which deals with the history of the hereditary neuroses of the Royal Family of Spain for a period of 250 years (1449-1700), showing how an insane and neuropathic taint in the ancestors has resulted in bringing about in the offspring various physical and mental defects and degeneracies, including epilepsy, imbecility (intellectual and moral), mania, melancholia, hypochondriasis, hysterical insanity, &c., and which the "fierce light that beats on a throne" has revealed to contemporary writers and observers. These genealogical tables and data tell their own tale.

[8] Traite des Degenerescences, 1857-60. [9] L'heredite dans les Maladies du Systeme Nerveux, Paris, 1886. [10] Degenerescence Sociale et Alcoalisme, Paris, 1895. [11] Uber Nervösen Famalien, 1884. [12] La Famille Névropathique, Paris, 1894. [13] An excellent summary by Lombroso himself is given in the Twentieth Century Practice of Medicine, Vol. XII., on Nervous and Mental Diseases, 1898. [14] The Jukes : A Study in Crime, Pauperism, Disease, and Heredity, New York, 1877. [15] On the Hereditary Nature of Crime, Journal of Mental Science, 1870. [16] A Treatise on Insanity in its Medical Relations, 1883, p. 179. [17] Sanity and Insanity, London, 1890, pp. 153-4. [18] The Blot upon the Brain : Studies in History and Psychology, 2nd edit., 1893, pp. 151-64.

## Figures and Tables

**Figure f1:**
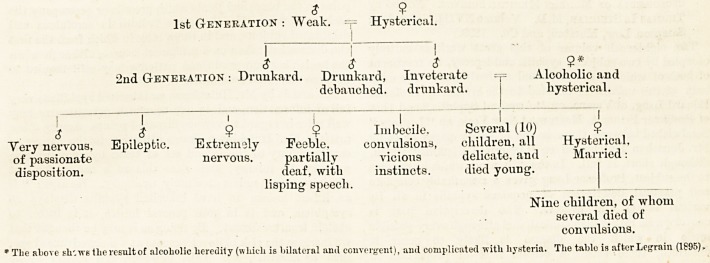


**Figure f2:**